# Visual outcome of cataract surgery in a tertiary care teaching hospital

**DOI:** 10.12669/pjms.38.4.5163

**Published:** 2022

**Authors:** Murtaza Sameen Junejo, Rebecca, Fahad Feroz Shaikh, Naimatullah Siyal

**Affiliations:** 1 Dr. Murtaza Sameen Junejo, FCPS, Department of Ophthalmology, Isra University Hospital, Hyderabad, Pakistan; 2 Dr. Rebecca, MBBS, Department of Ophthalmology, Isra University Hospital, Hyderabad, Pakistan; 3 Dr. Fahad Feroz Shaikh, FCPS, FRCS (GLASGOW), FCPS(VR), Department of Ophthalmology, Isra University Hospital, Hyderabad, Pakistan; 4 Prof. Dr. Naimatullah Siyal, MCPS, MS, Department of Ophthalmology, Isra University Hospital, Hyderabad, Pakistan

**Keywords:** Cataract, Clinical Audit, Phacoemulsification, Visual Acuity

## Abstract

**Objectives:**

To review the visual outcomes after cataract surgery in a tertiary care teaching hospital, Isra University Hyderabad.

**Methods:**

This retrospective clinical study was carried out for six months at Isra University Hyderabad from December 2020-May 2021. Total patients included were 982. The data comprised of patients who underwent cataract surgery from Sept 2018 – Aug 2020. Individuals over 40 years were included and who returned for out-patient appointments. Visual acuity before and six weeks after cataract surgery were noted and categorized according to World Health Organization criteria (i-e Good, Moderate and Poor).. Data analysis was done with the help of SPSS version 22.0.

**Results:**

Out of 982 patients who underwent Phacoemulsification at Isra University Hospital, the operated eyes were 483 right and 499 left respectively. Meanwhile, 966 had good visual acuity six weeks after the surgery, while 16 had moderate visual acuity noted after six weeks.

**Conclusion:**

Clinical audit of cataract surgeries by measuring visual acuity postoperatively is an excellent approach in improving the outcomes and maintaining the good care facilities at a tertiary care hospital.

## INTRODUCTION

Worldwide blindness is mainly caused by cataract and in Pakistan it alone is liable for 51.5% of blindness, consistent with Pakistan’s National Blindness and Visual Defect Survey 2002-03.^1^ A treatment for the cataract being the surgical extraction which usually is followed by a lens implantation. A Phacoemulsification is the micro incision suture less procedure for cataract removal worldwide and in developing countries like Pakistan, an extracapsular cataract extraction (ECCE) is also less commonly done.

Recent advances in technology of cataract extraction have made the life of everyone very comfortable. Ophthalmologists now offer patients high quality of lens implants. Following cataract removal, the implantation of intraocular lenses that provide simultaneous near and far correction is now routinely done.^2^ Due to bilateral cataracts 22 million people are blind within the whole world which is predicted to rise to 33 million by 2023.Half of all global blindness is cataract-induced and that is reversible. Safe and predictable cataract surgery outcomes are noticed only within the developed world; the rates of post-operative blindness are surprising within the developing world. Cataract surgery may also cause serious complications that can lead to blindness. In developing countries still the old technique of cataract extraction is being followed i-e couching.^3^-^5^

Clinical audit may be a tool, used not only to watch quality of services provided by doctors but also tells us whether we do it correctly up to the rules or not. The World Health Organization (WHO) has recommended that post-operative visual outcome after cataract surgery should be good (6/6-6/18) in 80% of cases. At the Layton Rahmatullah Benevolent Trust (LRBT) Eye Hospital, Lahore, a charity health center with a high surgical volume, Malik and colleagues reviewed the visual outcome of cataract surgery.^4^ Good visual outcome after correction was seen in 69.9% eyes. The authors audited the results of cataract surgery at the Isra University Hospital in Hyderabad to work out the extent to which WHO standards are being met.

## METHODS

This retrospective clinical audit was done over a six month period from December 2020- May 2021, and comprised data of patients who underwent cataract surgery from September 2018 to August 2020 after approval from Ethical Research Committee (ERC) Isra University, Hyderabad(IUH/ASST Dean(CS)/05/30). It had been limited to individuals aged over 40 years who had cataract surgery, and who returned for out-patient appointments. Patients excluded were those that had surgery for traumatic cataracts, or who had other ocular procedures like trabeculectomy or associated diabetic retinopathy and age related degeneration, performed at the time of cataract extraction. Visual acuity before and after surgery, and findings on examination were noted upto six weeks postoperatively. Keratometry (k1+ K2) and Amplitude scan (A-Scan) were performed to calculate intraocular lens (IOL) power using SRK T/ II formulae. Systemic comorbidities, mainly diabetes mellitus, hypertension, and ischemic heart disease were ruled out. Topical and/or peribulbar anaesthesia was used before commencement of a surgery. Injection xylocaine 2% was given in the inferotemporal quadrant and for topical proparacaine 0.5% eye drops were used. Cataract extraction technique used was phacoemulsification, along with implantation of intraocular lens (IOL). Post-operatively, topical moxifloxacin eye drops and steroidal anti-inflammatory medications were advised. Hypertonic saline 5% was given only to selected patients with striate keratopathy. Post-operative follow-up was done at day one, after one week, four weeks and six weeks respectively. Data analysis was done with help of SPSS version 22.0. Classification of visual acuity before and after surgery was done by using the WHO guidelines: Good outcome = 6/6-6/18; Borderline= <6/18 - 6/60 and Poor= <6/60.

## RESULTS

Total 982 patients had their cataract surgery at Isra Hospital, out of which there were 537 (54.7%) females and 445(45.3%) males having total mean of ±1.55 with SD ±.498 (Table-I & II). All patients completed their follow-up criteria. The eyes operated were 483(49.2) right eyes and 499 (50.8) left eyes having total mean value of ±1.51 and SD ±.500(Table-II). The Visual Acuity before surgery was mean of ± 2.39 with SD of ±.487 and after 06 weeks of surgery was ±1.02 and SD was ±.127 shown in Table-II. Preoperative VA was moderate in 61.3% patients andpoor in 38.7% patients. Meanwhile, VA after 06 weeks of phacoemulsification was good in 966 (98.4%) and moderate in 16 (1.6 %) of patients according to WHO criteria (Table-III).

**Table-I:** Total number of patients.

	Frequency	Percent
Male	445	45.3
Female	537	54.7
Right Eye	483	49.2
Left Eye	499	50.8

**Table-II:** Mean and Standard deviation of VA Before and After Cataract Surgery.

	Gender	Eyes	VA before surgery	BCVA after 06 weeks
n = total number	982	982	982	982
missing	0	0	0	0
mean	±1.55	±1.51	±2.39	±1.02
standard deviation	.498	.500	.487	.127

**Table-III:** VA before surgery.

	Frequency	Percent
<6/18 – 6/60 (Moderate)	602	61.3
<6/60 (Poor)	380	38.7
Best corrected VA 06 weeks after surgery		
6/6 – 6/18 (Good)	966	98.4
<6/18 – 6/60 (Moderate)	16	1.6
<6/60 (Poor)	0	0

## DISCUSSION

According to WHO database; cataract surgery is one among the foremost commonly performed ophthalmic surgeries worldwide, and its frequency will increase due to environmental and lifestyle changes in upcoming years. As cataract technique and technology advances, one has to demonstrate improving outcomes. To the best of our knowledge such a study with detailed surgical outcomes has not been conducted in our population.^6^,^7^

Overall, Pre-operative BCVA, consistent with WHO guidelines, was moderate in 602 (61.3%) patients and poor in 380 (38.7%) compared with 43% reported by the United Kingdom Cataract National Dataset Electronic Multicentre Audit (CNDEMA).^8^ There is a much bigger correlation of systemic co-morbidity with cataract surgery. Many systemic conditions can dramatically reduce potential for VA after surgery. The prevalence of preoperative recorded co-pathology is a smaller amount than those published in CNDEMA which was 71.5%. The common pre-operative ocular co-morbidity found in CNDEMA was proliferative diabetic retinopathy, glaucoma, corneal degeneration and age- related macular degeneration (ARMD).^8^ In contrast, we did not include the patients which had systemic co-morbidity in this study.^9^,^10^

In our study surgery was performed by highly qualified surgeons. In our tertiary care hospital, the peribulbar anesthesia is the most common route for anaesthesia in contrast with the NEON and PORT studies where the topical anaesthesia was the standard one, followed by peribulbar blocks that were given occasionaly.^8^,^9^

The source of data collection was manual as compared to electronic method of data collection done at various centres of NHS hospital which helps in systemic evaluation of various diseases record.^10^,^11^ It has been observed that akinetic anaesthesiamay reduce posterior capsule rupture (PCR) rates. The superiorty of anesthesia is not mentioned in literature in terms of PCR.^12^ In our study, Superiorty of akinetic anaesthesia over kinetic one was not observed. Cataract surgery in microophthalmic eye has significant results as studied in Al-Ibrahim eye hospital.^13^

It is observed that superior limbal incisions induce much more corneal astigmatic change than temporal incisions due to against the rule astigmatism.^13^-^17^ We did not calculate the amount of astigmatism in our study.

Another study conducted at Singapore National eye centre revealed significance of femtosecond assisted laser cataract surgery, which revealed better visual outcomes at 6 weeks.^10^,^13^ On the contrary, constellation machine was used in all surgeries at our hospital.

By doing clinical audit we calculate some important facts that are usually missed like steps of surgery, instruments used and time taken.^18^-^21^ So, for the betterment of surgical outcomes, audit and re audit should be conducted.^22^-^25^

Postoperative VA was recorded for 982 (100%) eyes. After cataract surgery, 966 (98.4%) eyes had good VA compared with pre-operative measurements. There have been 16(1.6%) eyes of patients who’s VA was moderate according to WHO criteria. The most common reason for moderate BCVA was posterior capsule rupture and cystoids macular oedema.

### Limitations of this study:

We did not calculate surgically induced astigmatism, Corneal degenerations, systemic co-pathology, post-operative retinal detachment and vitrous loss. BCVA was checked on snellen chart instead of LogMAR, which is the standard one used commonly due to its reliability. We also did not include patients with diabetic retinopathy and age related macular degeneration and time taken during surgeries.

## CONCLUSION

The surgeries performed at tertiary care hospital had a good outcome as they were performed by highly skilled surgeons. Such audit can be helpful in improving surgeons surgical skills, frequent visits prevents patients from devastating complications like endophthalmitis and also it improves the quality of small incision sutureless surgery.

### Authors’ Contribution:

**JMS:** Conceived, designed and did statistical analysis & editing of manuscript, is responsible for integrity of research.

**JMS, R, SFF:** Did data collection and manuscript writing.

**SN:** Did review and final approval of manuscript.
